# Assessment Tools for Measuring Health Literacy and Digital Health Literacy in a Hospital Setting: A Scoping Review

**DOI:** 10.3390/healthcare12010011

**Published:** 2023-12-20

**Authors:** Eline M. Dijkman, Wouter W. M. ter Brake, Constance H. C. Drossaert, Carine J. M. Doggen

**Affiliations:** 1 Department of Health Technology and Services Research (HTSR), Technical Medical Centre, University of Twente, 7522 NB Enschede, The Netherlands; e.m.dijkman@isala.nl (E.M.D.);; 2 Department of Surgery, Isala Hospital, 8025 AB Zwolle, The Netherlands; 3 Department of Psychology, University of Twente, 7522 NB Enschede, The Netherlands; c.h.c.drossaert@utwente.nl; 4 Clinical Research Center, Rijnstate Hospital, 6815 AD Arnhem, The Netherlands

**Keywords:** health literacy, ehealth literacy, digital health literacy, assessment tool, instrument, hospital

## Abstract

Assessment of (digital) health literacy in the hospital can raise staff awareness and facilitate tailored communication, leading to improved health outcomes. Assessment tools should ideally address multiple domains of health literacy, fit to the complex hospital context and have a short administration time, to enable routine assessment. This review aims to create an overview of tools for measuring (digital) health literacy in hospitals. A search in Scopus, PubMed, WoS and CINAHL, following PRISMA guidelines, generated 7252 hits; 251 studies were included in which 44 assessment tools were used. Most tools (57%) were self-reported and 27% reported an administration time of <5 min. Almost all tools addressed the domain ‘understanding’ (98%), followed by ‘access’ (52%), ‘apply’ (50%), ‘appraise’ (32%), ‘numeracy’ (18%), and ‘digital’ (18%). Only four tools were frequently used: the Newest Vital Sign (NVS), the Short Test of Functional Health Literacy for Adults ((S)TOFHLA), the Brief Health Literacy Screener (BHLS), and the Health Literacy Questionnaire (HLQ). While the NVS and BHLS have a low administration time, they cover only two domains. HLQ covers the most domains: access, understanding, appraise, and apply. None of these four most frequently used tools measured digital skills. This review can guide health professionals in choosing an instrument that is feasible in their daily practice, and measures the required domains.

## 1. Introduction

Every day, many patients visit their physician in, or are admitted to, the hospital. These patients usually need to read specific instructions about their visit beforehand, make decisions about treatments during their visit, and self-manage their disease after they have left the hospital. The hospital setting is complex: patients receive various health information from many different health professionals in a short period of time. Nowadays, hospital length of stay is increasingly reduced, as is the number of hospital visits [1]. As a consequence, health professionals have less time and opportunities to provide information about treatment and care and self-management becomes even more important. After a hospital admission patients receive instructions and advice which can have major consequences if not adhered to. Misunderstood information about potential complications after discharge may lead to serious consequences. 

Studies have shown that there is a considerable gap between the information provided by health professionals and what patients need or can understand [2]. Especially, patients with limited health literacy have difficulties with understanding and processing health information. Health Literacy is defined as “the degree to which individuals have the ability to find, understand, and use information and services to inform health-related decisions and actions for themselves and others” [3,4]. It is estimated that almost half (47%) of Europeans have limited health literacy [5]. Having limited health literacy is associated with more hospitalizations [6,7] and a longer hospital length of stay [8,9,10]. For example, in pancreato-biliary cancer surgery the length of stay was 13.5 vs. 9 days, respectively, for patients with and without limited health literacy [11], and for colorectal surgery this difference was 5 vs. 3.5 days [9]. Additionally, poor patient self-care, insufficient adherence to medication, a higher risk of post-surgical complications and even a higher mortality rate [12,13,14] were associated with limited health literacy. For example, in colorectal surgery, the number of postoperative complications differed significantly between patients with and without limited health literacy (43.5% vs. 24.3%) [9].

Current digital developments in hospitals, and the transition from care given in hospitals to care given in the home situation require also digital health literacy skills of patients such as operating devices, navigating on the internet and formulating questions for health professionals in e-consultation [15]. Digital health literacy, as defined by the World Health Organization, is the ability to seek, find, understand, and appraise health information from electronic sources and apply the knowledge gained to address or solve a health problem. Therefore, scholars have emphasized the need for health professionals to also gain insight into the level of digital health literacy of their patients [16].

In order to support patients with limited (digital) health literacy, and to reduce these negative consequences and resulting health disparities, health professionals in hospitals should adapt their communication and education to the needs of patients. Avoiding medical terms, using pictures and animations, referring to or helping with reliable digital resources and using the teach back-method, could help patients to understand the provided health information [17,18]. Yet, to be able to adapt their communication to the (digital) health literacy level of their patients, health professionals first need to recognize and identify patients with limited (digital) health literacy. 

Recognizing patients with limited (digital) health literacy can be difficult because patients are ashamed and therefore often do not spontaneously admit they have difficulties understanding the given information [9]. Moreover, health professionals often experience a high workload and as a result have limited time to identify a low (digital) health literacy level of patients. The hospital context makes this even more difficult, since patients often talk to many different care professionals in a relatively short period of time. Addressing patients’ health literacy cannot only solely depend on health professionals’ estimations or ‘gut-feelings’ but requires measurement and dialogue [19]. Unfortunately, (digital) health literacy is not often routinely measured in a hospital setting and studies have shown that patients’ (digital) health literacy level is often overestimated [19,20]. Health professionals have expressed a need for support in recognizing low (digital) health literacy, adapting communication [21]. Assessment tools for measuring (digital) health literacy level could be helpful to identify patients with limited (digital) health literacy, but should fit in the limited time available by health professionals.

Currently, there are various assessment tools available for measuring (digital) health literacy. These tools are usually not developed for use in the hospital setting. Often, administration time is rather long whereas available time in hospitals is short. Some tools are rather complex, and due to their physical or mental state, hospitalized patients may not be able to fill in elaborate questionnaires or answer questions. Therefore, more information is needed about which tools are useful in a hospital context. 

Existing reviews on available instruments have been conducted, but these do not focus on the hospital context [22,23,24,25,26], or focus on specific diseases or patient groups [27,28] such as cardiovascular diseases, on targeted departments, such as the emergency department [29], or on health literacy on the organizational level [30]. To our knowledge, no review about tools to assess (digital) health literacy in patients in the hospital context is available. To consider whether tools are suitable for health professionals in hospitals, insight is needed into various characteristics of the tools, such as administration time and mode of administration (self-administered or by professional), and whether the tool consists of self-reported questions or rather of an objective assessment. Moreover, the assessment tools vary with regard to the covered domains of health literacy. Six important domains can be distinguished [2]. First, patients need to be able to find and obtain (*access*) health information. Second, patients need to *understand* the health information and, third, to *appraise* the information, for example to determine if the information is reliable and if it relates to their personal situation. Fourth, to communicate with health professionals, patients have to be able to *apply* the health information to make decisions to maintain and improve health. Fifth, patients need *numeracy* skills in order to look after their health, for example to manage their diets, and to take appropriate medicine doses. And finally, the current digital developments in hospitals like patient portals and telemonitoring require the *digital skills* of patients [31]. 

Assessment of (digital) health literacy can enable hospitals to raise staff awareness of limited (digital) health literacy and provide tailored interventions based on patients’ level of (digital) health literacy. Dependent on the patients’ situation and specific department, one domain may be more important to assess than another. Unfortunately, routine assessment of (digital) health literacy of patients in hospital is not yet common. One reason for this may be lack of suitable instruments. Therefore, the aims of this current review are

- To create an overview of existing assessment tools for measuring (digital) health literacy of patients in the hospital;
- To investigate the characteristics of these tools (objective vs. self-reported, mode of administration, and administration time) and the domains that are covered by the tools (access, understand, appraise, apply, numeracy and digital skills)
- To examine to what extent studies have investigated the routine assessment of (digital) health literacy in daily clinical hospital practice.

## 2. Methods

A scoping review was conducted following the Preferred Reporting Items for Systematic Reviews and Meta-Analyses (PRISMA) guidelines [32]. A search in the databases’ Scopus, PubMed, Web of Science and CINAHL was conducted to identify eligible studies. The search string consisted of three search components combined by the Boolean operator AND, each containing a list of synonyms combined by the Boolean operator OR. The following key words were included in the search string: (digital) health literacy, assessment tool, and healthcare professional (Appendix A). A search string for each database was developed with support of an information specialist and can be found in Appendix B. All articles published before January 2023 were considered for eligibility. Duplicates of articles were removed. 

### 2.1. Eligibility 

Articles were included when they met the following inclusion criteria: (1) an assessment tool to measure (digital) health literacy was applied, and (2) participants were adult patients or familial caregivers, and (3) (digital) health literacy was measured in a hospital setting and (4) the article was published in English or Dutch. Excluded were (1) studies in which the used (digital) health literacy assessment tool measured only numeracy, or only literacy, mental literacy or oral literacy; (2) non-empirical studies; and (3) studies that used (digital) health literacy assessment tools that were developed only for specific patient groups (e.g., diabetes), and studies in which (digital) health literacy was only used to describe a population characteristic. 

### 2.2. Study Selection

**Figure 1:**
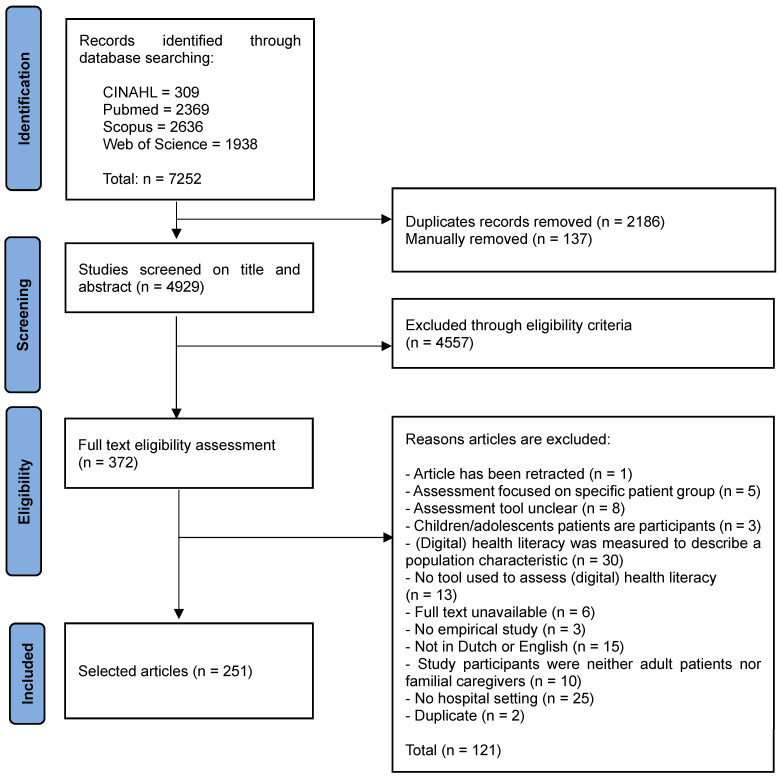
PRISMA flowchart of the search process.

Two researchers (ED, WB) independently screened all studies for initial eligibility on the title and abstract (The PRISMA flow chart in Figure 1 summarizes the results of the search process). Differences were solved through consensus meetings. If there still was disagreement, a third and fourth researcher (CJMD, CHCD) were consulted to reach consensus. Studies included after the title and abstract screening were further assessed for eligibility through full-text reading by both ED and WB. Disagreements were resolved by discussion and, if no agreement could be reached, the third and fourth researchers were consulted.

### 2.3. Data Extraction

First, data were extracted of all the included studies regarding the year of publication, assessment tool, setting, type and number participants. Secondly, characteristics of each assessment tool were extracted, including administration time, mode of administration, objective and self-reported questions or items. Besides these characteristics, the domains measured by each assessment tool were ascertained. (Digital) health literacy can be summarized through six domains: *access*, *understand*, *appraise*, *apply*, *numeracy* and *digital*. In order to consistently assess each tool, the domains of five random tools were determined and discussed by the overall project group (ED, WB, CJMD, CHCD). After this first step, two researchers (ED, WB) determined independently for each assessment tool which domains were measured. If there was disagreement, the third and fourth researcher were consulted to reach a consensus. 

## 3. Results

The PRISMA flow chart in Figure 1 summarizes the results of the search process. Our search generated 7252 articles. After duplicates were removed, 4929 were screened on title and abstract for eligibility, resulting in 4557 excluded articles. For the remaining 372 articles the full text was screened; 121 were excluded, mostly because (digital) health literacy was measured only for the purpose to describe a population characteristic (*n* = 30), no hospital setting (*n* = 25), not published in English or Dutch (*n* = 15), the article did not use an assessment tool for measuring (digital) health literacy (*n* = 13) or study participants were neither adult patients nor familial caregivers (*n* = 10). The remaining 251 articles were included in the analysis and the results of assessment tools that measured (digital) health literacy in a hospital setting are presented. 

### 3.1. Included Studies

The 251 articles were selected. Especially in recent years, there has been an increase in number of publications: 157 (63%) articles were published in the last 5 years (2018–2022). Most studies aimed to measure the incidence of health literacy, for example in patients with diabetes, cancer and cardiac illness, or to determine an association (digital) between health literacy and another component, such as complications and length of hospital stay. The number of patients included in the studies varied broadly between 8 and 5611 patients. 

### 3.2. Assessment Tools

In total, 44 different assessment tools for measuring (digital) health literacy were identified (Table 1). These 44 assessment tools were developed between 1991 and 2022. Tools that were most often used were the Newest Vital Sign (NVS) (*n* = 53 articles), the short Test of Functional Health Literacy for Adults ((S)TOFHLA) (*n* = 34), the Health Literacy Questionnaire (HLQ) (*n* = 32) and the Brief Health Literacy Screener (BHLS) (*n* = 28). Many of the identified assessment tools (25 out of 44) were used only occasionally, in only three or even fewer studies. Of the 44 assessment tools, fourteen were short or revised versions of an earlier developed assessment tool. 

### 3.3. Characteristics of the Assessment Tools

The 44 assessment tools varied considerably in administration time, mode of administration (interview vs. self-report), type of assessment (objective vs. self-reported) and in the number of items or questions (Table 1). Of the 44 assessment tools, 24 (55%) did not specify the administration time. Twelve tools (27%) reported an administration time of <5 min. The SILS, BHLS, (S)BHLS and REALM-SF take only one minute to administer, in contrast to the HLS-EU-Q47, which takes over twenty minutes, the longest administration time of all tools. The mode of all assessment tools differed: some instruments were to be applied as an interview, in other instruments participants were asked to fill out a questionnaire or to perform a test. More than half of all assessment tools included only self-reported questions *n* = 25 (57%), meaning that participants are asked to self-assess their own skills, for example by indicating how easy or difficult they consider various tasks. Examples of these are the BHLS, SILS and HLQ. Only 17 (39%) tools used objective assessments to assess a patients’ health literacy. Examples of these are the REALM, in which patients are asked to read a list of words aloud to the health professionals, or the NVS, where patients have to answer six questions about a nutrition label. Regarding the number of items of the tool, the most concise assessment tool consists of a single item (SILS and (S)BHLS), compared to the most extensive tool that consists of 82 items or questions (Health LiTT). 

### 3.4. Domains Measured by the Assessment Tools

The domains *access*, *understanding*, *appraisal*, *application*, *numeracy* and *digital*, which were covered by the assessment tools, are described in Table 2. Almost all tools address the *understanding* domain (98%). The *access* domain was covered by 20 (52%) tools, followed by 22 (50%) for *apply*, 14 (32%) for *appraise*, 8 (18%) for *numeracy* and 8 (18%) *digital skills*. The *digital* domain was added for the first time in 2006 in the assessment tool the eHEALS. 

Regarding the number of domains included in the tools, eight assessment tools (18%) included only one domain, namely *understanding*, such as the REALM, SILS and PHLKS. All other tools addressed at least two (*n* = 11 tools), three (*n* = 12) or four domains (*n* = 9). Only three, the eHEALS, ehils and DHLI, included 5 of the 6 domains. None of the assessment tools measured all six domains. 

### 3.5. Routine Assessment of (Digital) Health Literacy in Daily Practice in Hospitals

Out of the 251 included studies, only 4 studies reported about the routine assessment of (digital) health literacy in hospitals [111,150,254,284]. In the first study, the BHLS, a questionnaire consisting of three self-reported items, was used by 800 hospital and clinic patients and administered by nurses during routine clinical care. It demonstrated adequate reliability and validity to be used as a health literacy measure [150]. In the second study using the BHLS, health literacy was measured in 23,186 adult patients. The authors concluded that nurses in hospital and clinics adopted the new process quickly and reported it as beneficial for patient education discussions. The hospital setting had a rapid uptake of the BHLS and demonstrated sustained completion rates of more than 90% between November 2010 through April 2012 [111]. The third study [284] determined the feasibility of incorporating BHLS into EMR. Overall, nurses felt the screening was acceptable and useful. Nevertheless, some comments were noted: questions were repetitive, the patient did not understand questions or were annoyed, and nurses felt patients may not answer honestly. The last study used the REALM [254], a word recognition and pronunciation test consisting of seven items, was performed in a tertiary care academic medical center. In 1455 inpatients from nine representative floor units, the short version of the REALM was used to measure health literacy. The authors concluded that a routine health literacy assessment can be feasible and successfully implemented into the nursing workflow and electronic health record of a major academic medical center [254].

## 4. Discussion

When health professionals are aware of the (digital) health literacy level of their patients, health care delivery can be adapted to the needs of these patients and negative patient outcomes may be prevented. However, in hospitals the routine assessment of (digital) health literacy of patients is not common, possibly because of lack of (knowledge about) suitable instruments. This is the first scoping review that provides an overview of (digital) health literacy assessment tools used in the hospital setting. In total, 44 assessment tools were identified. Of all assessment tools, 27% reported an administration time of less than 5 minutes and 57% used self-reported questions. Almost all assessment tools addressed the domains of *understanding* (98%), followed by *access* (52%), *apply* (by 50%), *appraise* (32%), *numeracy* (18%) and *digital skills* (18%). Only four studies described routine use of (digital) health literacy in daily clinical practice in a hospital. 

Despite growing interest for the topic of (digital) health literacy in the last decade, our review revealed that relatively few studies (*n* = 251) have examined (digital) health literacy in the specific hospital context. Thereby, more than half of these studies were published in the last five years, indicating an increasing interest for the topic. No fewer than 44 assessment tools were identified. However, 4 four tools, Newest Vital Sign, the short Test of Functional Health Literacy for Adults, the Brief Health Literacy Screener, and the Health Literacy Questionnaire, were frequently used, while 18 (41%) assessment tools were used in only one study. A strength of the Newest Vital Sign and Brief Health Literacy Screener is their relatively short administration time of <5 min. Yet, for the Newest Vital Sign reading skills are required of patients when using this assessment tool, also when the instrument is administered by a health care provider. In addition, the NVS, BHLS and the (S)TOFHLA measure only two domains. Only the HLQ include four domains: *access*, *understand*, *appraise* and *apply*. None of these four tools measure the *digital skills*, which is a serious disadvantage given the current digitalization in hospital care, along with tools as patient portals, e-consultations, electronic patient reported outcome measurements (ePRO’s), and devices for (home) monitoring are increasingly being used.

The assessment tools differed widely in terms of characteristics and in the domains that are assessed. With respect to administration time, almost a third of the tools took 5 min or less. In hospital care, a short administration time is crucial, because of the high workload and short time available for consultations. Thereby, also patients are not motivated to fill in long surveys [310]. 

Both self-reported (57%) and objective (39%) assessment tools were found in our review. Only one tool, the eHLA, included both self-reported and objective questions. Depending on the specific clinical practice, there may be a preference for an objective or self-reported assessment. Moreover, patients often need reading skills to complete these assessments. The NVS, an objective assessment tool, was used most often. Another review identified the NVS as a practical instrument to quickly assess health literacy in the absence of a more comprehensive health literacy instrument [31]. However, the acceptability of self-reported instruments for patients is probably higher because it feels less like a test [150]. 

With respect to the domains, almost all tools address the domain *understanding* (98%), followed by *access* (52%), *apply* (50%), *appraise* (32%), *numeracy* (18%) and *digital* (18%). None of the assessment tools covered all six domains of (digital) health literacy. Only three instruments addressed five of the six domains and almost a third included only one domain. Thus, although new instruments have been developed over the last years, there is not yet one assessment tool which includes all the domains. This indicates the difficulty of developing an assessment tool which can easily be used in a hospital by health professionals, which includes all the necessary domains and still does not require a long administration time. 

Another notable finding was that only eight instruments address the digital domain. This in an era where increasingly, digital devices are implemented in hospitals, such as telemonitoring, apps, and patient portals. These innovations can contribute to a high patient engagement and improve patient outcomes. Unfortunately, to use these innovations patients need adequate digital skills. Thus, knowledge of the (lack of) digital skills of a patient may help the health professional in the decision to offer digital devices or not. 

It turns out to be difficult to develop one tool that meets all the requirements of a suitable assessment tool to be used in daily clinical practice. A relatively new assessment tool, the Conversational Health Literacy Assessment tool (CHAT), provides insight into HL skills without using a long questionnaire. This assessment tool consists of 10 questions which support health professionals to engage in conversations with patients about specific health literacy strengths and challenges. The conversational approach of the CHAT promotes open communication instead of measuring what a patient is not capable of. Further evaluation of the utility and feasibility in daily practice is necessary and the digital component was still lacking. 

To our knowledge, this is the first review including studies that assess the effect of the routine screening of (digital) health literacy in the hospital. Despite the importance of identifying patients with low (digital) health literacy, we found that it is still not common in daily practice. Only four studies [111,150,254,284] reported the routine assessment and registration of health literacy. Despite the importance, only four studies reported on the routine assessment of health literacy in the hospital setting. In the literature, various challenges are mentioned that can explain the paucity of studies on the topic. These include a lack of knowledge about suitable instruments and about the prevalence and consequences of low (digital) health literacy [311], a lack of belief in the benefits of assessing health literacy [311] or being afraid that discussing this delicate issue may cause shame or have a negative impact on patient engagement, trust and willingness to seek healthcare services [312], a lack of skills [312] and a lack of time [150]. 

Nevertheless, the four studies that were conducted all concluded that the routine assessment of health literacy is feasible, suggesting that the aforementioned challenges can be overcome. However, various limitations should be taken into account when interpreting the results of these studies. First, two of the four studies took place in the same hospital [111,150]. Second, one of the four studies was conducted in a university hospital where the educational level of patients was relatively high, limiting its generalizability [111]. Third, even though nurses in one of the studies were positive about feasibility in the questionnaire, this questionnaire was only completed by 27% [284]. 

Studies have reported reluctance among health care professionals towards the routine assessment of (digital) health literacy, because health literacy screening can contribute to patients feeling stigmatized and ashamed of having limited (digital) health literacy [313]. Recognizing the (digital) health literacy level of patients is important for health professionals to adapt their communication to the level of each individual patient. There are advantages and disadvantages for measuring health literacy in clinical practice. First, using an assessment tool is important because there are differences between (digital) health literacy estimation by health professionals and the actual (digital) health literacy level of patients [314]. Also, patients recognize the importance of literacy in their healthcare and most are comfortable with literacy assessment [315]. On the other hand, patients can feel ashamed and stigmatized having low (digital) health literacy. 

Future research should focus on the actual use of assessment tools in daily practice and explore the facilitators and barriers of nurses in identifying patients with low (digital) health literacy. Moreover, more attention should be paid on cultural and linguistic diversity on health literacy assessment. In general, knowledge and education of available assessment tools for health professionals creates awareness and prevents negative outcomes for patients [20]. There are several training programs available for health professionals on health literacy resulting in positive outcomes on for example knowledge and skills [316]. These training programs are diverse, varying in duration, frequency and content. Some training programs also include specific education about assessment of health literacy. 

However, we do not yet know if nurses in hospitals are educated, and if so, how they receive training in identifying patients with low (digital) health literacy. Insight into facilitators and barriers in using assessment tools gives the opportunity to improve communication between nurses and their patients. 

This review has some notable strengths: first, the search string consists of multiple keywords to broaden the scope of the search in several databases. In addition, the data extraction was performed by two researchers, after the first five assessment tools were discussed by all four researchers to reach consensus. A limitation is that the review only included articles written in English and Dutch. Moreover, this review did not measure the quality of the included articles because the main aim was to find available assessment tools for (digital) health literacy. 

## 5. Conclusions

This review provides an overview of available assessment tools for (digital) health literacy used in hospitals. Thereby, it provides insight into the variation in characteristics and domains included in the assessment tools. Ideally, such a tool should be able to assess several domains of health literacy including digital skills, and have a short administration time, so it can be routinely used. Currently, there is not one assessment tool that meets all these requirements and does not cause shame. We may question whether it is even possible to develop such a tool that fits all requirements. The results of this review can be used to guide health professionals in hospitals in choosing an assessment tool that is feasible in their own daily clinical practice and measures the domains that are most relevant in the particular situation. Future research should examine how the barriers for routine assessment of (digital) health literacy in the hospital can be overcome.

## Figures and Tables

**Table 1:** Characteristics of the (digital) health literacy assessment tools.

	(Digital) Health Literacy Tool ^A^	Publ. YEAR	Full Name	Short Description	Items, Questions or Domains	Time ^B^ (min.)	Mode of Administration	Objective/Self- Reported	Number of Included Studies ^C^	References of the Included Studies
1	NVS [33]	2005	Newest Vital Sign	Ice cream nutrition label comprehension test	6 questions, including 5 closed questions and open ended questions	3–4	Interview	O	53	[19,34,35,36,37,38,39,40,41,42,43,44,45,46,47,48,49,50,51,52,53,54,55,56,57,58,59,60,61,62,63,64,65,66,67,68,69,70,71,72,73,74,75,76,77,78,79,80,81,82,83,84,85]
2	(S)TOFHLA [86]	1999	Short Test of Functional Health Literacy in Adults	Test	40 items: 36 closed type reading comprehension, 4 calculations	7–12	Interview/Self-reported	O	34	[39,46,60,62,66,84,87,88,89,90,91,92,93,94,95,96,97,98,99,100,101,102,103,104,105,106,107,108,109,110,111,112,113,114]
3	HLQ [115]	2013	Health Literacy Questionnaire	Questionnaire	44 items, 9 domains: (1) 4 on feeling understood and supported by healthcare providers, (2) 4 on having sufficient information to manage health, (3) 5 on actively managing my health, (4) 5 on social support for health, (5) 5 on the appraisal of health information, (6) 5 on the ability to actively engage with healthcare providers, (7) 5 on navigating the healthcare system, (8) 5 on the ability to find good health information, (9) 5 on understanding health information well enough to know what to do	7.5	Interview/Self-reported	S	32	[87,115,116,117,118,119,120,121,122,123,124,125,126,127,128,129,130,131,132,133,134,135,136,137,138,139,140,141,142,143,144,145]
4	BHLS [146]	2004	Brief Health Literacy Screener	Questionnaires	Three items with 5-point Likert type scale	1	Interview/Self-reported	S	28	[52,103,111,138,147,148,149,150,151,152,153,154,155,156,157,158,159,160,161,162,163,164,165,166,167,168,169,170]
5	REALM [171]	1991	Rapid Estimate of Adult Literacy in Medicine	Word recognition and pronunciation test	66 items: general list of medical words in increasing levels of difficulty	2.5	Interview	O	17	[50,98,99,172,173,174,175,176,177,178,179,180,181,182,183,184,185]
6	FCCHL [186]	2008	Functional Communicative Critical Health Literacy	Questionnaire	14 items: 5 on Functional, 5 on Communicative, 4 on Critical	NS	Interview/Self-reported	S	16	[108,187,188,189,190,191,192,193,194,195,196,197,198,199,200,201]
7	eHEALS [202]	2006	eHealth Literacy Scale	Questionnaire	8 items with 5-point Likert-type scale	NS	Self-reported	S	12	[127,203,204,205,206,207,208,209,210,211,212,213]
8	TOFHLA [214]	1995	Test of Functional Health Literacy for Adults	Test	67 items: 50 reading comprehension closed type, and 17 numerical ability test	22	Interview/Self-reported	O	9	[114,174,185,215,216,217,218,219,220]
9	SAHL (S&E) [221]	2010	Short Assessment of Health Literacy—Spanish & English	Word recognition and comprehension test	18 items: For every item, the respondent gets presented two words, they have to choose which one is meaningfully related to the term	2–3	Interview/Self-reported	O	8	[41,55,215,222,223,224,225,226]
10	HLS-EU-Q47 [227]	2013	European Health Literacy Survey Questionnaire	Questionnaire	47 items: 22 items on the healthcare domain, 13 items on the disease prevention domain, 11 on the health promotion domain. The second section consists of sociodemographics /economics, health behavior, health status, health service use, community participation. Four-point Likert scale	20–30	Interview/Self-reported	S	7	[65,81,228,229,230,231,232]
11	HLS-EU-Q16 [233]	2014	16 items short European Health Literacy Survey Questionnaire	Questionnaire	Sixteen items selected from the HLS-EU-Q47, assessing the same domains. Four-point Likert scale	10	Interview/Self-reported	S	6	[203,234,235,236,237,238]
12	HELIA [239,240]	2014/ 2020	Health Literacy for Iranian Adults/Health Literacy Instrument for Adults	Questionnaire	33 items: 4 on reading comprehension, 6 on accessing, 7 on understanding, 4 on evaluation, 12 on decision making and behavior	NS	Self-reported	S	6	[241,242,243,244,245,246]
13	(S)BHLS [247]	2008	Short Brief Health Literacy Screener	One question	1 item with 5-point Likert response	1	Interview/Self—reported	S	5	[18,80,92,97,248]
14	REALM-R [248]	2003	Rapid Estimate of Adult Literacy in Medicine—Revised	Word recognition and pronunciation test	8 items: General list of medical words in increasing levels of difficulty, revised	2	Interview	O	5	[39,46,158,249,250]
15	REALM-SF [251]	2007	Rapid Estimate of Adult Literacy in Medicine—short form	Word recognition and pronunciation test	7 items: general list of medical words in increasing levels of difficulty, shortened	1	Interview	O	4	[45,252,253,254]
16	SILS [255]	2006	Single Item Literacy Screener	One question	1 item: 5-point Likert-type scale and categorized as inadequate or adequate	1	Interview/Self-reported	S	4	[39,40,60,133]
17	eHLQ [256]	2018	eHealth Literacy Questionnaire	Questionnaire	35 items: (1) 5 on using technology to process health information, (2) 5 on the understanding of health concepts and language, (3) 5 on the ability to actively engage with digital services, (4) 5 on feeling safe and in control, (5) 5 on motivation to engage with digital services, (6) 4 on access to digital services and (7) 6 on digital services that suit individual needs	7	Self-reported	S	4	[87,136,257,258]
18	HLS-SF12 [259]	2019	Short Form Health Literacy Questionnaire	Questionnaire	12 items in 4 dimensions: assessing (items 1, 5, 9), understanding (items 2, 6, 10), appraising (3, 7, 11), and applying (items 4, 8, 12), which can further be categorized into three domains: healthcare, disease prevention, and health promotion.	3–5	Interview/Self-reported	S	4	[260,261,262,263]
19	SAHLSA_50 [264]	2006	Short Assessment of Health Literacy for Spanish Adults	Word recognition and comprehension test	50 items: for every item, the respondent gets presented two words, they have to choose which one is meaningfully related to the term	3–6	Interview/Self-reported	O	3	[40,74,225]
20	BRIEF [265]	2009	Brief Health Literacy Screening Tool	Questionnaire	3 items of BHLS + 1 item with 5-point Likert-type scale	2	Interview/Self-reported	S	3	[133,266,267]
21	HLS(-14) [268]	2013	Health Literacy Scale -14	Questionnaire	14 items: three subscales including 5 functional literacy items, 5 communicative literacy items, and 4 critical literacy items.	NS	Interview/Self-reported	S	3	[73,269,270]
22	Health LiTT [271]	2009	Health Literacy Assessment Using Talking Touchscreen Technology	Computer-based test	82 items, 3 domains: reading comprehension closed type; identify and interpret information in graphs/tables; numerical operations	18	Computer based & Self-reported	O	2	[193,195]
23	(S)MHLS [272]	2012	Short-form Mandarin Health Literacy Scale	Test	11 items: 8 reading tests, 3 numerical tests, multiple choice closed type response	NS	Self-reported	O	2	[273,274]
24	HELP [275]	2013	Health Education Literacy of patients with chronic musculoskeletal diseases	Questionnaire	18 items: (1) 6 on comprehension of medical information, (2) 5 on the application of medical information, (3) 7 on communicative competence in provider interactions	NS	Self-reported	S	2	[275,276]
25	AHLS [277]	2014	Adult Health Literacy Scale	Test	22 items on drug use and health information, and a figure for pointing out the location of organs in the human body: 13 yes–no, 4 fill-in-the-blanks, 4 multiple-choice, 2 matching questions on a scale	NS	Self-reported	O	2	[213,278,279]
26	(S)KHLT [280]	2017	Short Form of the Korean Functional Health Literacy Test	Test	8 items: 4 numeracy, 4 reading comprehension	NS	Self-reported	O	2	[281,282]
27	EBHLS [283]	2014	Expanded Brief Health Literacy Screening Tool	Questionnaire	3 items of BHLS + 2 items with 5-point Likert-type scale	2	Interview	S	1	[284]
28	PHLKS [285]	2008	Public Health Literacy Knowledge Scale	Test	17 items: general health knowledge statements, true-or-false response	NS	Self-reported	O	1	[286]
29	(S)Health LiTT [287]	2014	Short Form Health Literacy Assessment Using Talking Touchscreen Technology	Computer-based test	14 items: 6 closed type, 6 understanding/interpretation, 2 numerical operations	NS	Computer based & Self-reported	O	1	[288]
30	EHILS [289]	2012	Everyday Health Information Literacy Screening Tool	Questionnaire	10 items with 5-point Likert-type scale	NS	Self-reported	S	1	[290]
31	(S)KHLS [291]	2013	Korean Health Literacy Scale short form	Test	12 items: 7 comprehension and numeracy, 5 health-related	10	Interview/Self-reported	O	1	[292]
32	HeLMS [293]	2013	Health Literacy Management Scale	Questionnaire	29 items: (1) 4 on patient attitudes towards their health, (2) 4 on understanding health information, (3) 4 on social support, (4) 3 on socioeconomic considerations, (5) 4 on accessing GP healthcare services, (6) 3 on communication with health professionals, (7) 3 on being proactive, (8) 4 on using health information. Five-point Likert scale	NS	Self-reported	S	1	[293]
33	HLS [294]	2013	Health Literacy Scale	Questionnaire	25 items, 4 domains: 5 items on accessing (range: 5–25), 7 items on understanding (range: 7–35), 8 items on appraising (range: 8–40), 5 items on applying (range: 5–25) health information. Five-point Likert scale	NS	Interview/Self-reported	S	1	[295]
34	HLS-EU-Q6 [233]	2014	6 items short-short European Health Literacy Survey Questionnaire	Questionnaire	6 items selected from the HLS-EU-Q47, assessing the same domains. Four-point Likert scale	NS	Interview/Self-reported	S	1	[196]
35	TSOY-32 [296]	2016	Turkish Health Literacy Scale-32	Questionnaire	32 items: 4 point Likert response	NS	Interview	S	1	[297]
36	(S)DHLI [15]	2017	Short Digital Health Literacy Instrument	Questionnaire	9 items on the handling of web 2.0 tools	NS	Self-reported	S	1	[298]
37	eHLA [299]	2018	Electronic Health Literacy Assessment Toolkit	Toolkit consisting of seven tools	44 items, divided over 7 tools: (1) 10 on functional HL, (2) 9 on self-assessed HL, (3) 5 on familiarity with health and disease, (4) 6 on knowledge of health and disease, (5) 6 on digital familiarity, (6) 4 on digital confidence, (7) 4 on digital incentives	NS	Self-reported	O/S	1	[257]
38	READHY [300]	2019	Readiness and Enablement Index for Health Technology	Questionnaire	65 items: 13 dimensions from the heiQ (4), HLQ (2), and eHLQ (7). Four-point Likert-type response	NS	Self-reported	S	1	[300]
39	MMHLQ [301]	2017	Mandarin Multidimensional Health Literacy Questionnaire	Questionnaire	20 items, 5 domains: acquiring, understanding, assessing, applying and communication. Four-point Likert scale.	NS	Self-reported	S	1	[302]
40	NLP [303]	2014	Natural Language Processing tools	Computer-based	Computer-based tool to translate healthcare information.	NS	Computed-based	NS	1	[303]
41	HLS-(19)-COM-P-Q11 [304]	2022	Communicative Health Literacy Survey Questionnaire	Questionnaire	11 items selected from HLS, focused on communication. Four-point Likert scale.	NS	Self-reported	S	1	[304]
42	RIHLA [305]	2021	Rapid Independent Health Literacy Assessment	Questionnaire	15 items on general health knowledge. Three answers including “I don’t know”	NS	Self-reported	O	1	[305]
43	ComprehENotes [306]	2018	Comprehension Electronic health record Notes	mHealth	Health tool based on artificial intelligence to annotate medical documents into understanding information	NS	Self-reported	O	1	[307]
44	CHAT [308]	2018	Conversational Health Literacy Assessment tool	Questionnaire	10 open-ended questions across five domains: 4 on supportive relationships, 2 on health information access, 2 on current health behaviors and 2 on barriers and support on health promotion.	NS	Self-reported	S	1	[309]

(A) (digital) health literacy assessment tool; (B) average administration time in minutes; (C) number of studies including the assessment tools; NS = Not specified in included studies.

**Table 2:** Domains of the (digital) health literacy assessment tools.

(Digital) Health Literacy Tool	Publ. Year	Access ^A^	Understand ^B^	Appraise ^C^	Apply ^D^	Numeracy ^E^	Digital ^F^	Ref.
1. AHLS	2014	X	X	X				[277]
2. BHLS	2004	X	X					[146]
3. (S)BHLS	2008		X					[247]
4. BRIEF	2009	X	X					[265]
5. CHAT	2018	X	X		X			[308]
6. ComprehENotes	2018	X	X				X	[306]
7. (S)DHLI	2017	X	X	X	X		X	[15]
8. EBHLS	2014	X	X		X			[283]
9. eHEALS	2006	X	X	X	X		X	[202]
10. EHILS	2012	X	X	X	X		X	[289]
11. eHLA	2018	X	X				X	[299]
12. eHLQ	2018	X	X				X	[256]
13. FCCHL	2008	X	X	X	X			[186]
14. Health LiTT	2009		X		X	X		[271]
15. (S)Health LiTT	2014		X		X	X		[287]
16. HELIA	2014/2020	X	X	X	X			[239,240]
17. HeLMS	2013	X	X		X			[293]
18. HELP	2013		X		X			[275]
19. HLS	2013	X	X	X	X			[294]
20. HLS(-14)	2013	X	X		X			[268]
21. HLS-(19)-COM-P	2022		X		X			[304]
22. HLS-EU-Q47	2013	X	X	X	X			[227]
23. HLS-EU-Q16	2014	X	X	X	X			[233]
24. HLS-EU-Q6	2014	X	X	X				[233]
25. HLS-SF12	2019	X	X	X	X			[259]
26. HLQ	2013	X	X	X	X			[115]
27. (S)KHLS	2013		X			X		[291]
28. (S)KHLT	2017		X			X		[280]
29. (S)MHLS	2012		X			X		[272]
30. MMHLQ	2017	X	X		X			[301]
31. NLP	2014	NA	NA	NA	NA	NA	NA	[303]
32. NVS	2005		X			X		[33]
33. PHLKS	2008		X					[285]
34. READHY	2019		X	X	X		X	[300]
35. REALM	1991		X					[171]
36. REALM-R	2003		X					[248]
37. REALM-SF	2007		X					[251]
38. RIHLA	2021		X		X			[305]
39. SAHLSA_50	2006		X					[264]
40. SAHL (S&E)	2010		X					[221]
41. SILS	2006		X					[255]
42. TSOY-32	2016	X	X	X	X			[296]
43. TOFHLA	1995		X			X		[214]
44. (S)TOFHLA	1999		X			X		[86]

(A) Access refers to the ability to seek, find and obtain health information; (B) understand refers to the ability to comprehend the health information that is accessed; (C) appraise describes the ability to interpret, filter, judge, and evaluate the health information that has been accessed; (D) apply refers to the ability to communicate and use the information to make decisions to maintain and improve health; (E) numeracy refers to the ability to perform calculations in a health setting, for example, doses in medication intake; (F) digital refers to the ability to seek, find, understand, and appraise health information from electronic sources and apply the knowledge gained to address or solve a health problem (NA) Not Applicable.

## Data Availability

No new data were created or analyzed in this study. Data sharing is not applicable to this article.

## Abbreviations

NVS	Newest Vital Sign
HLQ	Health Literacy Questionnaire
(S)TOFHLA	Short Test of Functional Health Literacy in Adults
BHLS	Brief Health Literacy Screener
REALM	Rapid Estimate of Adult Literacy in Medicine
FCCHL	Functional Communicative Critical Health Literacy
eHEALS	eHealth Literacy Scale
SAHL (S&E)	Short Assessment of Health Literacy—Spanish & English
TOFHLA	Test of Functional Health Literacy for Adults
HLS-EU-Q47	European Health Literacy Survey Questionnaire
HLS-EU-Q16	European Health Literacy Survey Questionnaire 16 items short
HELIA	Health Literacy for Iranian Adults/Health Literacy Instrument for Adults
(S)BHLS	Short Brief Health Literacy Screener
REALM-R	Rapid Estimate of Adult Literacy in Medicine—Revised
REALM-SF	Rapid Estimate of Adult Literacy in Medicine—short form
SAHLSA_50	Short Assessment of Health Literacy for Spanish Adults
SILS	Single Item Literacy Screener
eHLQ	eHealth Literacy Questionnaire
HLS-SF12	Short Form Health Literacy Questionnaire
BRIEF	Brief Health Literacy Screening Tool
HLS(-14)	Health Literacy Scale -14
Health LiTT	Health Literacy Assessment Using Talking Touchscreen Technology
(S)MHLS	Short-form Mandarin Health Literacy Scale
HELP	Health Education Literacy of patients
AHLS	Adult Health Literacy Scale
(S)KHLT	Short Form of the Korean Functional Health Literacy Test
EBHLS	Expanded Brief Health Literacy Screening Tool
PHLKS	Public Health Literacy Knowledge Scale
(S)Health LiTT	Short Form Health Literacy Assessment Using Talking Touchscreen Technology
EHILS	Everyday Health Information Literacy Screening Tool
(S)KHLS	Korean Health Literacy Scale short form
HeLMS	Health Literacy Management Scale
HLS	Health Literacy Scale
HLS-EU-Q6	6 items short European Health Literacy Survey Questionnaire
TSOY-32	Turkish Health Literacy Scale-32
(S)DHLI	Short Digital Health Literacy Instrument
eHLA	Electronic Health Literacy Assessment Toolkit
READHY	Readiness and Enablement Index for Health Technology
MMHLQ	Mandarin Multidimensional Health Literacy Questionnaire
NLP	Natural Language Processing tools
HLS-(19)-COM-P-Q11	Communicative Health Literacy Survey Questionnaire
RIHLA	Rapid Independent Health Literacy Assessment
ComprehENotes	Comprehension Electronic health record Notes
CHAT	Conversational Health Literacy Assessment tool

## Appendix A. Search Terms

### Abbreviations

**1**	**2**	**3**
“Health literacy”, “health competence”, “eHealth literacy”, “e-health literacy” “digital literacy”.	“Assessment” “assessment tool”, “questionnaire”, “scale”, “screening tool”, “survey”, “literacy screen”, “measure”, “tool”.	“Nurses”, “health care professional”, “healthcare professional”, “health care provider”, “healthcare provider”, “health care worker”, “healthcare worker”, “caregiver”, “health personnel”, “physician”, “clinical staff”, “health staff” “patient-professional communication”.

## Appendix B. Search Strings

1. PubMed search string

((((((((((((nurs*[Title/Abstract]) OR (health care professional*[Title/Abstract])) OR (healthcare professional*[Title/Abstract])) OR (health care provider*[Title/Abstract])) OR (healthcare provider*[Title/Abstract])) OR (health care worker*[Title/Abstract])) OR (healthcare worker*[Title/Abstract])) OR (caregiver*[Title/Abstract])) OR (health personnel*[Title/Abstract])) OR (physician*[Title/Abstract])) OR (clinical staff*[Title/Abstract])) OR (health staff*[Title/Abstract])) OR (patient professional communication[Title/Abstract])

AND

((((((Health literacy[MeSH Terms])) OR (Health literacy[Title/Abstract])) OR (Health competence[Title/Abstract])) OR (Ehealth literacy[Title/Abstract])) OR (E-health literacy[Title/Abstract])) OR (Digital literacy[Title/Abstract])

AND

((((((((Assessment[Title/Abstract]) OR (Assessment tool[Title/Abstract])) OR (Questionnaire*[Title/Abstract])) OR (Scale[Title/Abstract])) OR (Screening tool[Title/Abstract])) OR (Survey[Title/Abstract])) OR (Literacy Screen[Title/Abstract])) OR (Measure*[Title/Abstract])) OR (Tool*[Title/Abstract])

2. Scopus search string

TITLE-ABS-KEY ((nurs* OR professional OR provider) AND (“health literacy” OR “digital literacy”) AND (“assessment tool” OR questionnaire OR survey OR “literacy scale”))

3. Web of Science search string

TS = ((nurs* OR professional OR provider) AND (“health literacy” OR “digital literacy”) AND (“assessment tool” OR questionnaire OR survey OR “literacy scale”))

4. CINAHL search string

((MH “Health Literacy”) OR “health competence” OR ehealth OR “digital literacy”) AND (assessment OR MH “Questionnaires+” OR MH “Scales” OR screening tools OR MH “Surveys+”) AND MH “Nurses+”
